# Need Assessment of Existing Mentorship Program Among Undergraduate Medical Students: Experience From a Medical College in Chhattisgarh, India

**DOI:** 10.7759/cureus.47413

**Published:** 2023-10-21

**Authors:** Sunil Kumar Panigrahi, Gitismita Naik, Gouri K Padhy, Himel Mondal, Sudip Bhattacharya

**Affiliations:** 1 Community and Family Medicine, All India Institute of Medical Sciences, Deoghar, Deoghar, IND; 2 Community and Family Medicine, All India Institute of Medical Sciences, Kalyani, Saguna, IND; 3 Community and Family Medicine, All India Institute of Medical Sciences, Raipur, Raipur, IND; 4 Physiology, All India Institute of Medical Sciences, Deoghar, Deoghar, IND

**Keywords:** india, communication, curriculum, developed countries, professionalism, facilitator, competency based medical education, programme, mentorship, medical student

## Abstract

Introduction

While mentoring students during regular medical education has a long-standing tradition in many developed countries' medical schools, it has yet to become a standard practice in the majority of medical institutions, especially in the developing world, such as India. In institutions where mentoring programs are sparsely implemented, there is a lack of data regarding their assessment.

Methodology

This qualitative study involved two groups of students - nine undergraduate medical students (five male and four female) and 10 undergraduate medical students (six male and four female) who had at least three years of experience in the existing mentorship program at a tertiary care teaching hospital. We conducted two focused group discussions (FGDs) with these two groups of students using a guide, with FGDs lasting 45 and 50 minutes, respectively. We recorded the audio and it was transcripted to text. Thematic analysis of the transcripts from the 2 FGDs was conducted using Atlasti (Version 7.1.8) software to assess perceptions of the mentorship program.

Results

The content analysis of the discussions revealed two broad themes, namely “Current Functioning of the Programme” and “Suggestions for Improvement.” These themes were further divided into multiple domains and subdomains, providing a comprehensive overview of the study's findings. Although there is a consensus among students that the mentorship program is essential, the current operational framework still has limited confidence due to biases, fears, and misinformation among the students.

Conclusion

The ongoing medical curriculum imparts a vast amount of scientific knowledge within a limited timeframe, with practical application occurring primarily in the last three years of the academic curriculum and minimal emphasis on ethical practice, professionalism, effective communication, handling urgent health situations, and interacting with family members, underscores the genuine need for a structured mentorship curriculum for undergraduate medical students. To enhance the program's effectiveness, the active involvement of undergraduate students must address their specific needs.

## Introduction

Mentorship in medical education has a long history in medical schools across the world [1]. The advantages of mentoring a mentee or trainee medical undergraduate by a mentor or a senior faculty or a teacher are well documented and wide-ranging, like enhanced innovation, research encouragement, career progression, and enhanced learning experience [2,3]. The medical education system for undergraduates in developing countries like India is evidently fraught with multiple stressors ranging from peer pressure, immense study curriculum workloads, indifferent study or work environments, high parental expectations, ragging, and difficulties in adjusting to a new independent hostel life, etc. [4-6]. These factors are even more significant in the initial years of medical education, which brings along all lifestyle changes at once. Mentoring programs intend to provide a much-needed cushion in softening the transition [7]. A mentor here is a senior academician, who can show the way and counsel the junior person in dealing with difficult or confusing choices by using their own experience for the growth and development of the mentee [8].

Most of the available literature on mentorship is from medical schools of developed countries where the culture and context are very different from countries like India. Mentorship as a practice is also fairly new in the country. A critical examination of the ongoing mentorship programs is essential for critical course corrections of the ongoing mentorship programs which will not only foster improvement for these programs but also improve participation in the same. With this background, this study aimed to assess the perceived need and benefit of the existing mentorship program among undergraduate students.

## Materials and methods

Type and settings

This study employed a qualitative research design and was conducted at a tertiary care teaching hospital. The research focused on undergraduate medical students' perceptions of the existing mentorship program within the medical institute. This study was approved by the Institutional Ethics Committee of All India Institute of Medical Sciences, Raipur (AIIMSRPR/IEC/2020/323).

Sample

The study included two groups of undergraduate medical students. One group consisted of nine participants, with a gender distribution of five males and four females in each group. The second group of 10 participants had six males and four females. The participants were selected from two different semesters of undergraduate students, and all had been part of the existing mentorship program for a minimum of three years.

Data collection methods

**Figure 1:**
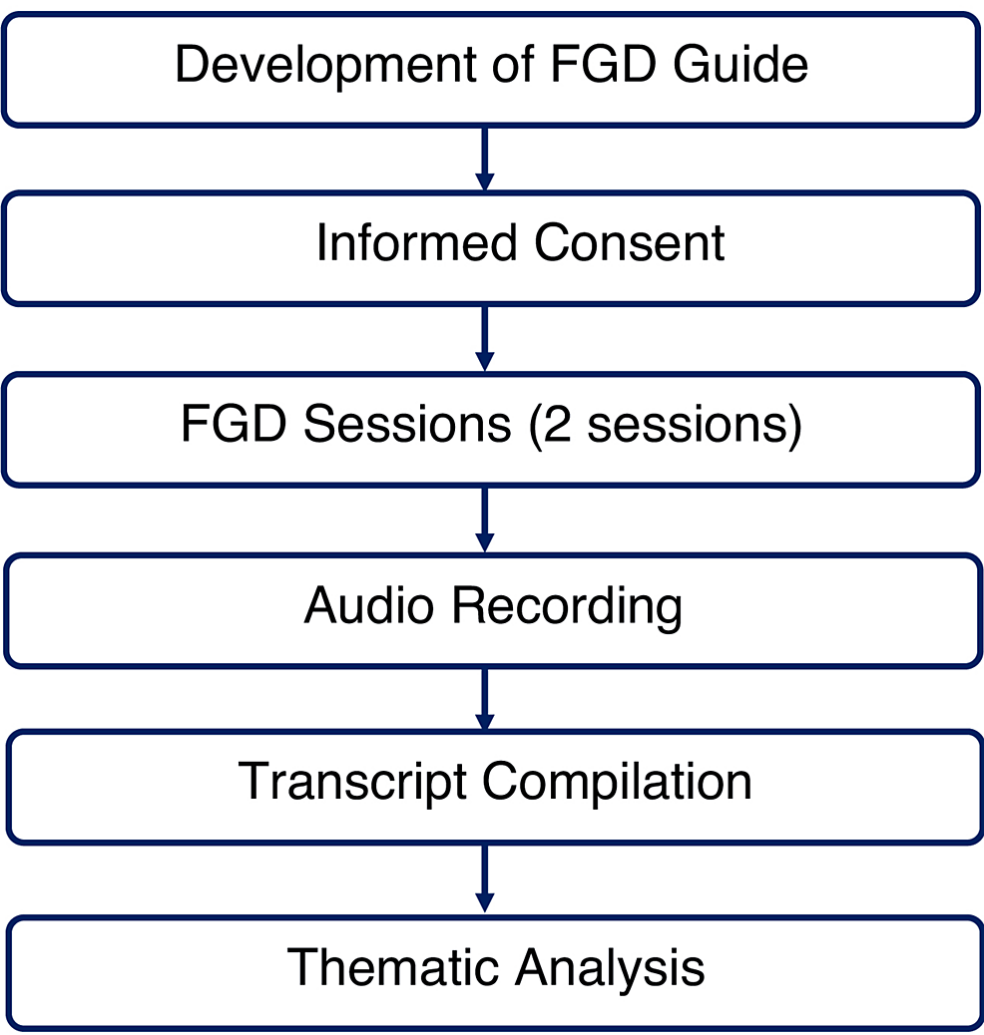
Brief study procedure FGD: Focused group discussion The figure is created by Dr. Himel Mondal for this manuscript

The primary data collection method used in this study was focused group discussions (FGDs). Two FGDs were conducted to gather insights into the participants' perceptions regarding the functioning, usefulness, and need for the existing mentorship program. The FGDs had a duration of approximately 45 and 50 minutes, respectively. The process is briefly described in Figure 1.

Data analysis

The textual data were thematically analyzed by Atlasti (https://atlasti.com) to generate themes, domains, and sub-domains. The result is presented with direct quotes.

## Results

Two FGDs were conducted among two batches of students separately to assess the perception of the functioning and usefulness of the mentorship program. The content analysis of the discussions revealed two broad themes as shown in Table 1.

**Table 1:** Themes, domains, and sub-domains identified during thematic analysis of the focused group discussions

Themes	Domains	Sub-domains
Current functioning of the program	Program status	Program optimism; Program pessimism; Critical turnaround
	Program perception	Fear; Prejudice; Misinformation; Unfelt need
	Program issues	Issue with initiation; Issue with process
	Program barriers	Barriers among students; Barriers among mentors; Environmental barrier
	Program need	Addressing felt need; Rapport building; Flexibility; Enabling environment
Suggestions for improvement	Suggestion for students	-
	Suggestion for mentors	-
	Suggestion for program	-

**Figure 2:**
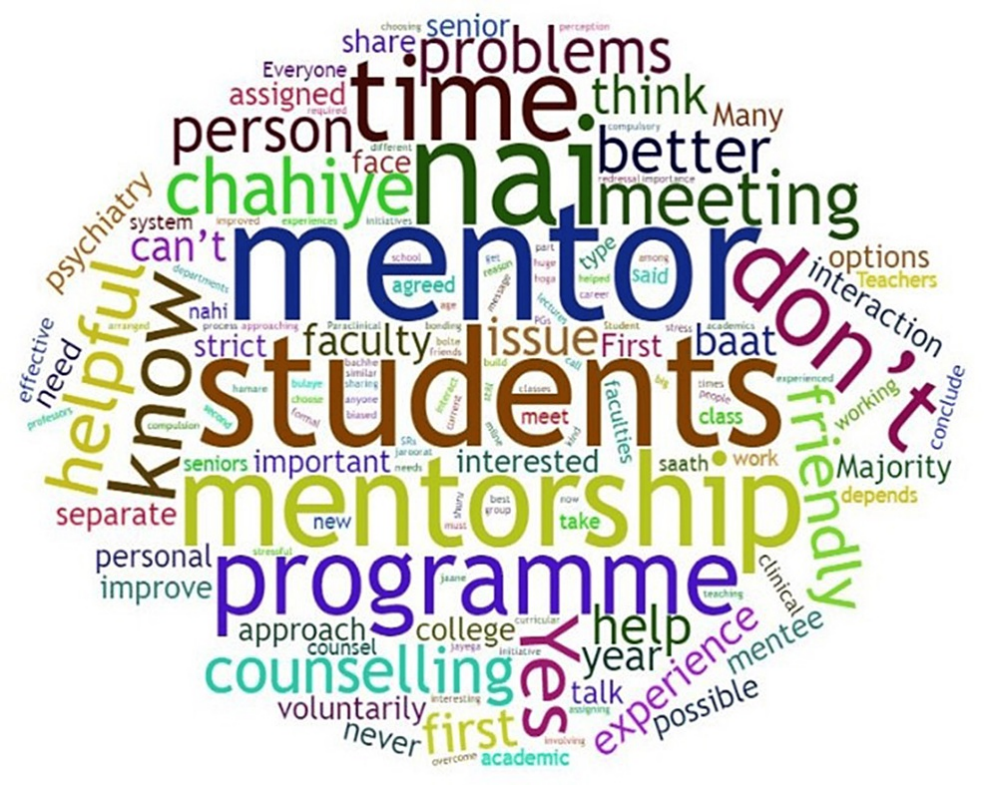
Word cloud made from the text transcript of the audio The figure was created by the first author for this manuscript

The word crunch analysis of FGDs (Figure 2) showed the words according to their frequencies in the text.

Program status

The discussion on the status of the ongoing mentorship program elicited radically divergent views when one of the participants stated, “Half of the students don’t even know about the existence of the program, it’s a failure.” However, another participant suggested, “The programme is useful. I met my mentor after a week of assigning; it was a stressful time for me. I wanted someone who had gone through a similar experience. Meeting my mentor was helpful. I met around 3 three times.” Some participants had a change of view during the course of mentorship, which was indicated by the following statement by a participant. These are critical turnaround moments during the process of mentoring a student. One of them said, “he (mentor) was able to understand me, acknowledged my problems, and helped me.” It can be concluded that, though the ongoing program needs improvements and is criticized for being ineffective, the program nevertheless is an important felt need among the students, many of whom were optimistic about the prospect of the mentorship program, if improved and implemented properly.

Program perception

One of the dominant perceptions among the students was fear of approaching or interacting with the teachers. One of the students stated, “Students don’t even ask doubts.” Another stated, “We feel, why bother a faculty for a small issue.” The fear may be because of the authority of a teacher, which prevails during academic discussions. Students feeling fearful is a reflection of the behavior that prevents them from asking questions or doubts freely during classroom discussions. Some of the students were unwilling to go to their mentors because of the previous adverse opinions of their academic seniors, who in a way influenced the decision-making of their juniors. Many of the students perceived teachers in some of the departments are busy and they have little time to devote compared to some other departments, where teachers are more amenable. There are prevailing prejudices among the students regarding the judgemental attitude of teachers, which was indicated when a student stated, “There should be friendly approach by the mentors. Many students feel the mentor will be judgemental & make a fixed bad opinion of them.”

Issues in the implementation of the program

The program faced hindrances during two phases of its implementation - the initiation phase and the continuation phase. The initial phase of the program of meeting with the mentor and starting the mentorship is the most vital cog in the success of the program. It is in this phase most of the rapport fails to materialize, which eventually culminates into an unsuccessful mentoring opportunity. One student stated that “most students just know that they have a mentor but don’t do anything beyond that.” Other factors that affect the initiation of the programs are lack of choice for the students to select their mentors, lack of interested and amenable mentors, lack of flexibility in meeting due to overburdening formality, and behavioral constraints among the students preventing them from approaching the mentor. The effect of successful initiation of the program has a long-term advantage over the success of the program as one of the statements by a student, “I met my mentor after a week of assigning; it was a stressful time for me. But, meeting my mentor was helpful. I met around 3 three times.”

The issues with the continuation of the program lie in the failure of rapport building, lack of role clarity, or understanding of the objectives of the program. During the course of the program, the lack of motivation to continue and the communication gap between the mentor or the administration and mentees also has adverse effects on the program. The statement by one of the students indicates these issues - “My mentor was changed, I did not meet him. If we don’t know the person at all, like mentors in new departments, where we don’t know anyone, the chances of interaction even if assigned are very low. Many times I feel nonclinical faculties or our teachers are better options.”

Program barriers

The barriers existing among the students were mostly behavioral factors like fear of approach, lack of motivation, and prejudice regarding the mentors. One of the students stated “I had two mentors, each was approachable. But I did not feel like sharing my problem with them.” Barriers among the mentors are both physical factors like lack of time due to a busy schedule and behavioral factors like strict disciplinarian attitude toward the students in academic classes or expectations from the students, which prevents students from discussing freely. One student opined, "Some teachers are very strict. Students don’t feel like going to them. Many of them are even scared to go.” The environmental barriers identified were administrative miscommunication or delayed communication about the timing of mentorship meetings and, the opinion of the academic seniors about some teachers, which influences the decision-making among the students.

The need for the program

**Figure 3:**
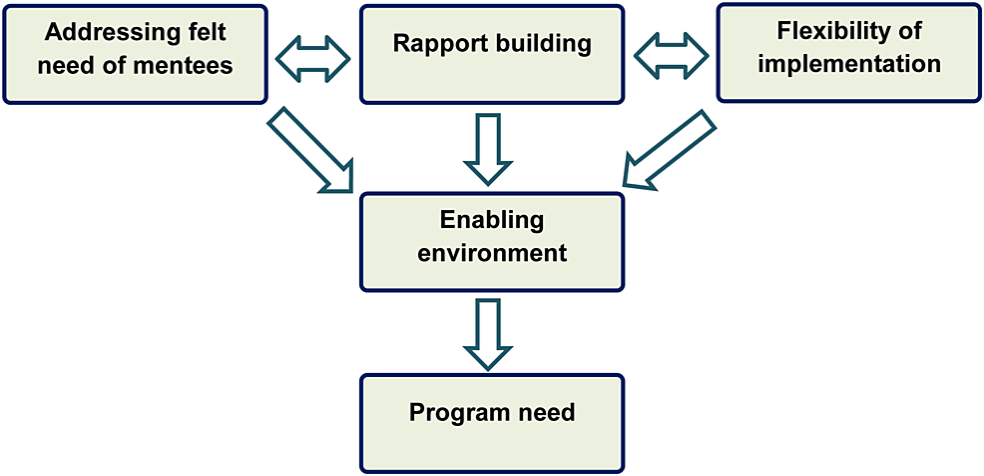
Suggested environment for a successful outcome of the mentorship program The figure was created by the first author and modified by Dr. Himel Mondal

The need for the program depends on the triad of addressing the felt need among the students or mentees, active efforts to improve rapport building in the initial phase of the program, and flexibility in implementing the program which will eventually create an enabling environment for a successful outcome of the mentorship program. Flexibility within these programs allows for personalized guidance tailored to the unique needs and goals of each mentee. Furthermore, by creating an enabling environment, mentorship programs ensure that mentees feel supported, encouraged, and empowered to seek guidance, ask questions, and take risks in their journey toward personal and professional success. In summary, a mentorship program that incorporates these themes (Figure 3) can have a profound impact on individual and organizational development.

Suggestions for improvement

Suggestions were contributed enthusiastically by the participants of FGDs. They suggested improvements on the part of students, teachers, and the mentoring environment. Suggestions for students were pertaining to efforts to allay fear while approaching the mentor and efforts to improve the chances of taking initiative first. One statement suggested, “There is something lacking among us also. If we start going, then maybe it will work better.” Suggestions for the mentors were improvement through fostering a friendly and amenable atmosphere during the discussion, taking the initiative to approach an introverted and shy mentee, and making individual efforts in rapport building. These will improve the mentor-mentee relationship with healthy participation from both sides. One of the students suggested that “Introverts have a hard time approaching. If for such people mentors could take the initiative.” Suggestions for the administrative aspect of the program included the creation of an enabling environment through a proactive and sensitive approach in improving the first meeting and rapport building among the mentor and mentees. There is a need for improved communication and reduced professional formality regarding the mentorship meetings. One student opined that “Simply putting up notices to meet the assigned teacher is not helpful. Our needs should also be looked into.”

## Discussion

In both India and abroad, many prestigious medical schools have established mentorship programs. However, there is a notable dearth of studies evaluating these programs and their effects in India. The limited research available underscores the significance of such programs. For instance, a study conducted at the University College of Medical Sciences in Delhi found that 97% of students and 80% of teachers believed that a mentorship program was a crucial necessity in their institution [9]. Mentors often refrained, assuming that mentees would take the first step, while mentees found it challenging to initiate contact with mentors who were often their teachers and sometimes senior. Another study reported that although the continuation of the mentoring process relies on mentee involvement, the initial contact should ideally be initiated by the mentor [10]. Similar observations emerged from this study.

Studies have also highlighted that mentors who make time for the mentoring process are perceived as effective mentors [9,11]. Finding a suitable time for interaction was a commonly recognized challenge in the process [12,13]. A systematic review of mentorship programs also identified the absence of a “protected time” as a barrier to a successful mentor-mentee relationship [14]. Participants who had dedicated time to mentoring viewed this commitment from their institutions as a positive signal of support [15]. Another study revealed that the perception among mentees that the mentor-mentee relationship was merely superficial and exploitative posed a significant challenge to the program unless actively addressed [16]. We also found concerns about potentially creating a negative impression on their teachers due to their interactions within the program.

Ssemata et al. reported that effective mentoring involves defining mentor roles, emphasizing mutual trust and respect, distinguishing mentor and supervisor roles, addressing challenges in mentor selection, and overcoming barriers through formalized programs and context-specific training [17]. Although challenges may exist for implementation [18], along with students, early career doctors benefit a lot from mentorship programs [19]. Hence, an optimum cooperation between mentor and mentee would make the program successful [20].

This study sheds light on a relatively underexplored area by examining the impact of mentorship programs in medical schools in India, where there is a notable dearth of research on the subject. The study offers insights into the specific challenges and barriers faced by both mentors and mentees, emphasizing the importance of mentor-initiated contact and the need for dedicated time for mentoring, which can inform the design and implementation of more effective mentorship programs. Furthermore, by highlighting the perception of mentorship as a cosmetic or exploitative relationship, the study underscores the significance of addressing these concerns for program success.

However, this study is not without limitations. Its findings are context-specific and may not be entirely applicable to other educational or professional settings. The study relies on self-reported data, which may be subject to respondent bias or social desirability bias. Additionally, the research predominantly focuses on qualitative data, which limits the ability to establish causal relationships or generalize findings. Future research should consider these limitations and aim to expand the scope of inquiry to provide a more comprehensive understanding of mentorship programs' impact and challenges.

## Conclusions

The support for the mentorship program is necessary for success and sustenance. There are many barriers that hinder a successful mentoring program, but most of these are amenable to change. Minor modifications in the program to facilitate its uptake and a fresher perspective or an open mind from mentors towards the mentees could help make the program more acceptable. Longitudinal follow-up of the program to examine and log the changes, improvements, and benefits can be a useful resource. This knowledge gap needs to be filled with more research that informs on our country’s context and also explores the various forms of mentor-mentee programs and their impact.
